# *Campylobacter fetus* spondylodiscitis during immunochemotherapy for non-Hodgkin's lymphoma

**DOI:** 10.1590/0037-8682-0801-2020

**Published:** 2021-03-08

**Authors:** Jéssica Santos Cunha, Fernando Franco Lafetá Queiroz, Rodrigo Juliano Molina, Leonardo Rodrigues de Oliveira

**Affiliations:** 1 Universidade Federal do Triângulo Mineiro, Hospital de Clínicas, Programa de Residência Médica em Clínica Médica, Uberaba, MG, Brasil.; 2 Universidade Federal do Triângulo Mineiro, Hospital de Clínicas, Disciplina de Doenças Infecciosas e Parasitárias, Uberaba, MG, Brasil.; 3 Universidade Federal do Triângulo Mineiro, Hospital de Clínicas, Serviço de Hematologia e Hemoterapia, Uberaba, MG, Brasil.

**FIGURE 1:**
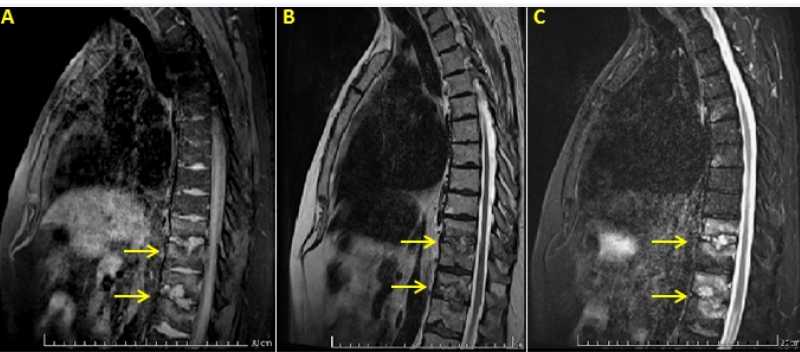
Magnetic resonance imaging with gadolinium infusion of the lumbar spine in T1 sequence **(A)**, T2 sequence **(B)** and short TI inversion recovery (STIR) mode **(C)**. The findings include diffuse disc edema and inflammation between T10 and T11 and T12 and L1 vertebral bodies (arrows), with no abscess or significant reduction in the height of the vertebral bodies.

A 58-year-old man presented with fever and backache, which he had begun experiencing 3 h before admission. Laboratory tests showed normal leukocyte (4.2×10^9^/L) and neutrophil (4.1×10^9^/L) counts, lymphocytopenia (85/mm³), and increased C-reactive protein level (23.1 mg/dL, normal up to 0.5 mg/dL). The patient was administered rituximabe for mantle cell non-Hodgkin's lymphoma following eight immunochemotherapy sessions. Contrast-enhanced computed tomography was undertaken for persistent backache, which revealed a lytic lesion in the T12 region. Nuclear magnetic resonance demonstrated changes that were aligned to spondylodiscitis (Figure 1). There was no evidence of lymphoma relapse. *Campylobacter fetus (C. fetus)* infection was confirmed using a mass spectrometer. Treatment was initiated with the administration of intravenous azithromycin (500 mg/day) and gentamicin (240 mg/day) for 14 days, followed by intravenous ertapenem (1 g/day) for an additional 14 days in an outpatient setting. Fever and back pain were managed in a constant and sustained manner.

Spondylodiscitis is the inflammation of the intervertebral discs and adjacent vertebral bodies. *Campylobacter* bacteria are rarely reported to be the cause of spondylodiscitis^1^. Spondylodiscitis is an uncommon infection and is related to risk factors such as diabetes mellitus, malnutrition, immunosuppression, neoplasms, renal failure, HIV infection, alcoholism, and gastrointestinal surgery^2^. Spondylodiscitis occurs predominantly by hematogenous dissemination of pathogens into the urinary tract, respiratory tract, or soft tissues^2^. Spondylodiscitis due to *C. fetus* is very rare; hence, its management and optimal treatment has not been identified^3^.
